# Influence of *FMO3* and *CYP3A4* Polymorphisms on the Pharmacokinetics of Teneligliptin in Humans

**DOI:** 10.3389/fphar.2021.736317

**Published:** 2021-08-26

**Authors:** Jin-Woo Park, Kyoung-Ah Kim, Jong-Min Kim, In-Hwan Park, Ji-Young Park

**Affiliations:** ^1^ Department of Clinical Pharmacology and Toxicology, Anam Hospital, Korea University College of Medicine, Seoul, South Korea; ^2^ Department of Neurology, Anam Hospital, Korea University Medical Center, Seoul, South Korea

**Keywords:** teneligliptin, FMO3, CYP3A4, genetic polymorphism, pharmacokinetics

## Abstract

Teneligliptin, a dipeptidyl peptidase-4 inhibitor, is used to treat type 2 diabetes mellitus. FMO3 and CYP3A4 metabolize teneligliptin into teneligliptin sulfoxide. This study examined the effects of *FMO3* (rs909530, rs1800822, rs2266780, and rs2266782) and *CYP3A4* (rs2242480) polymorphisms on teneligliptin pharmacokinetics at a steady state among 23 healthy participants administered 20 mg teneligliptin daily for 6 days. Subjects with *FMO3* rs909530, rs2266780, and rs2266782 polymorphisms exhibited a significant gene dosage-dependent increase in maximum steady-state plasma drug concentration (C_max,ss_) and area under the drug concentration vs time curve (AUC) (*p*<0.05). However, the C_max_ values significantly decreased but the AUC values did not significantly vary in subjects with *CYP3A4* polymorphism (rs2242480). These results suggest that *FMO3* and *CYP3A4* polymorphisms affect teneligliptin pharmacokinetics in humans. The findings of this study provide a scientific basis for the inter-individual variation in teneligliptin disposition.

## Introduction

Teneligliptin, which belongs to the family of dipeptidyl peptidase-4 inhibitors, is used to treat type 2 diabetes mellitus (T2DM). The absorbed fraction of teneligliptin is approximately 74%. The liver metabolizes 66–80% of teneligliptin into teneligliptin sulfoxide, while the kidney mediates the excretion of 20–34% of the drug (1–4). Flavin-containing monooxygenase 3 (FMO3) and cytochrome P450 3A4 (CYP3A4) are equally involved in teneligliptin metabolism in the liver (3).

Previous clinical and *in vitro* studies have elucidated the mechanism of teneligliptin metabolism using various inhibitors of FMO3 and CYP3A4, such as ketoconazole and methimazole (5,6). Ketoconazole and methimazole (FMO inhibitor) inhibit teneligliptin metabolism by 47.0 and 67.2%, respectively, in the human microsomes (7). Recent studies have demonstrated the roles of FMO3 (8,9) and CYP3A4 (10–12) in the disposition of their substrates. *FMO3* polymorphisms, including rs909530 (c.855C>T, N285N), rs2266782 (c.472G>A, E158K), and rs2266780 (c.923A>G, E308G), are reported to affect the pharmacokinetic characteristics of sulindac, a FMO3 substrate (8,9). Additionally, rs2242480 (g.20230G>A, IVS10 + G12A, and CYP3A4*1G) located in intron 10 is one of the most frequent genetic polymorphisms in the East Asian population and affects the pharmacokinetic characteristics of various drugs by up-regulating (13) or downregulating CYP3A4 activity (14,15). However, the underlying mechanisms have not been elucidated.

The effects of *FMO3* and *CYP3A4* on the pharmacokinetics of teneligliptin are unclear. Therefore, we hypothesized that *FMO3* and *CYP3A4* polymorphisms affect the pharmacokinetics of teneligliptin based on the currently known teneligliptin metabolism pathways. This study aimed to examine the potential effects of *FMO3* (rs909530, rs1800822, rs2266780, and rs2266782) and *CYP3A4* (rs2242480) polymorphisms on teneligliptin pharmacokinetics in humans.

## Material and Methods

### Subjects

In this study, 23 healthy male Korean volunteers were enrolled. Detailed physical examination, 12-lead electrocardiogram, vital parameters, and laboratory tests, including blood chemistry, hematology, and urine analyses, were performed to determine the health status of the volunteers. The exclusion criteria were as follows: history or evidence of hepatic, renal, gastrointestinal, or hematological pathologies; hepatitis B or C and human immunodeficiency virus infections; any other acute or chronic disease; and drug allergies. The participants were not allowed to consume drugs 2 weeks before or during the study period. The study protocol was approved by the Institutional Review Board of Korea University Anam Hospital (IRB no.2017AN0117). Written informed consent was obtained from all volunteers. All procedures were performed according to the Declaration of Helsinki and Good Clinical Practice guidelines.

### Study Design

The participants were administered 20 mg teneligliptin (Handok Inc., Seoul, Korea) daily for 6 days to reach a steady state. On day 6 post-drug administration, the serial blood samples were collected immediately before (0 h) and after 0.25, 0.5, 1, 1.5, 2, 3, 4, 6, 8, 10, 12, and 24 h of dosing. The blood samples were collected in ethylenediaminetetraacetic acid (EDTA) tubes (Vacutainer; Becton Dickinson, Franklin Lakes, New Jersey, United States) and centrifuged at 1977 g and 4°C for 15 min. The plasma samples were stored at −70°C until analysis. The participants were genotyped for FMO3 (rs909530, rs1800822, rs2266780, and rs2266782) and CYP3A4 (rs2242480) single-nucleotide polymorphisms (SNPs).

### *FMO3* and *CYP3A4* Genotyping

Genomic DNA was isolated from the peripheral leukocytes as described previously (Hoffmeyer et al., 2000; Cascorbi et al., 2001). The rs909530, rs1800822, rs2266780, and rs2266782 polymorphisms of FMO3 and the rs2242480 polymorphism of CYP3A4 were identified using pyrosequencing with a PSQ 96MA Pyrosequencer (Pyrosequencing AB, Uppsala, Sweden) (Kim et al., 2013). The details of the primers used for each *FMO3* and *CYP3A4* SNPs are described in Supplementary Table S1 (Park et al., 2021, Submitted).

### Bioanalysis

The teneligliptin and teneligliptin sulfoxide concentrations were measured as described previously (Park et al., 2020). Briefly, the sample was injected into a high-performance liquid chromatography system (Shiseido Co., Ltd, Japan) coupled with an API 4000 mass spectrometer (Applied Biosystems-SCIEX, MA, United States) equipped with a Capcell Pak C18 column (2.0 mm × 150 mm, 5 μm, Tokyo, Japan) and a guard column. The isocratic mobile phase was a mixture of acetonitrile (100%) and methanol (50%; diluted in distilled water) (1:1; v:v). The flow rate of the mobile phase was 0.25 mL/min. The mass spectrometer was equipped with an electrospray ionization source and operated in positive ion mode with multiple reaction monitoring. The mass transition ion pairs of teneligliptin and teneligliptin-d8 were selected as m/z 427.2→ 243.1 and m/z 435.2→ 251.3, respectively. Standard working solutions of teneligliptin (1,000, 500, 100, 50, 10, 5, and 2 ng/mL) were prepared by diluting the stock solution with blank plasma. A linear calibration curve of standard teneligliptin was established (*r* ^2^ = 0.9996).

### Pharmacokinetic Analysis

The pharmacokinetic parameters of teneligliptin and teneligliptin sulfoxide were determined using non-compartmental analysis with Phoenix^®^ WinNonlin^®^ software (version 8.0, Certara™, Princeton, United States). The minimum steady-state plasma concentration during the dosage interval (Cmin,ss), maximum steady-state plasma concentration during the dosage interval (C_max,ss_), and time to reach C_max,ss_ (T_max_) were estimated directly from the observed plasma concentration vs time plots. The total areas under the plasma concentration-time curves during the dosing interval (AUC_τ_) were calculated using the linear trapezoidal rule for 24 h after the final dose.

The t_1/2_ of teneligliptin was calculated as follows:

$${\text{t}}_{1/2}=\text{ln}2/{\text{K}}_{e}$$

The oral clearance (CL/F) of teneligliptin was calculated as CL/F = dose/AUC_τ_.

### Statistical Analysis

All data are expressed as mean ± standard deviation unless otherwise indicated. The differences were considered significant at *p* <0.05. The pharmacokinetic parameters among *FMO3* and *CYP3A4* genotypes were comparatively analyzed using one-way analysis of variance or Kruskal-Wallis test, followed by Tukey’s post hoc analysis after examining the normal distribution of the data. Genetic equilibrium and linkage disequilibrium were determined according to the Hardy-Weinberg equation using SNPalyzer version 9.0 (DYNACOM Co., Ltd., Yokohama, Japan). All statistical analyses were performed using the SAS statistical software package version 9.4. (SAS Institute, Cary, NC, United States).

## Results

### Demographic Data

In total, 23 healthy subjects were recruited in this study (age: 24.7 ± 2.3 years; height, 174.3 ± 3.7 cm; weight, 68.3 ± 6.7 kg). The frequencies of genotypes (*FMO3* and *CYP3A4*) and demographic data according to the genotypes are shown in Supplementary Table S2. The genotype was not significantly correlated with any of the covariates.

### Genetic Analysis of *FMO3* and *CYP3A4* Polymorphisms

The observed allele frequencies for *FMO3* rs909530, rs1800822, rs2266780, and rs2266782 polymorphisms were 0.391, 0.152, 0.239, and 0.239, respectively, whereas those for *CYP3A4* rs2242480 polymorphism were 0.261. The allele frequencies measured in this study did not deviate from the Hardy-Weinberg equilibrium (Table 1). *FMO3* rs2266780 and rs2266782, which were in complete linkage disequilibrium (*r* ^2^ = 1), exhibited a strong linkage disequilibrium with FMO3 rs909530 (*r* ^2^ = 0.4889; *p* < 0.05; Table 2).

**TABLE 1:** Effect of *FMO3* and *CYP3A4* polymorphisms on teneligliptin pharmacokinetic parameters.

**Parameter**	**Wild-type (W)**	**Heterozygous (H)**	**Homozygous mutant (M)**	**H and M**	***p*** **-value**		
					**W vs H vs M**	**W vs H and M**	HWE
*FMO3* (rs909530)	GG (*n* = 9)	GA (*n* = 10)	AA (*n* = 4)	GA, AA (*n* = 14)			0.9848
C_min,ss_ (ng/mL)	29.99 ± 7.80	34.36 ± 8.86	42.43 ± 12.09	36.66 ± 14	0.0964	0.1079	
C_max,ss_ (ng/mL)	259.44 ± 22.42	314.6 ± 61	367.25 ± 101.01	329.64 ± 74.43	0.016^ab,^*	0.0125*	
AUC_τ_ (ng·h/mL)	1939.62 ± 289.61	2527.31 ± 383.35	2763.76 ± 545.01	2594.86 ± 427.29	0.0019^ab,^*	0.0006*	
Half-life (h)	14.18 ± 3.17	14.56 ± 5.19	12.60 ± 2.05	14 ± 4.53	0.7214	0.9184	
CL/F (L/h)	10.52 ± 1.62	8.07 ± 1.18	7.42 ± 1.24	7.89 ± 1.19	0.0009^ab,^*	0.0002*	
*FMO3* (rs1800822)	GG (*n* = 17)	GA (*n* = 5)	AA (*n* = 1)	GA, AA (*n* = 6)			0.9580
C_min,ss_ (ng/mL)	32.18 ± 14.64	39.46 ± 14.33	38.80	39.35 ± 12.82	0.3094	0.1212	
Cmax, ss (ng/mL)	295 ± 62.07	319.6 ± 97.2	337	322.5 ± 87.23	0.7003	0.4101	
AUC_τ_ (ng·h/mL)	2235.82 ± 439.44	2678.08 ± 622.25	2385.35	2629.29 ± 569.24	0.2201	0.0948	
Half-life (h)	14.64 ± 4.4	12.15 ± 1.81	13.96	12.45 ± 1.78	0.4900	0.2558	
CL/F (L/h)	9.29 ± 1.89	7.78 ± 1.69	8.38	7.88 ± 1.53	0.2881	0.1167	
*FMO3* (rs2266780/rs2266782)	AA (*n* = 13)	AG (*n* = 9)	GG (*n* = 1)	AG, GG (*n* = 10)			0.8323
C_min,ss_ (ng/mL)	30.95 ± 8.42	39.30 ± 9.95	27.2	38.09 ± 8.42	0.1023	0.0787	
C_max,ss_ (ng/mL)	277.15 ± 52.45	320.22 ± 63.68	465	334.7 ± 75.5	0.0107^b,^*	0.0426*	
AUC_τ_ (ng·h/mL)	2087.98 ± 393.83	2673.3 ± 454.39	2581.15	2664.09 ± 429.39	0.0137^a,^*	0.0031*	
Half-life (h)	13.69 ± 2.78	14.83 ± 5.51	12.11	14.56 ± 5.27	0.7267	0.6157	
CL/F (L/h)	9.88 ± 1.79	7.66 ± 1.2	7.75	7.67 ± 1.13	0.0122^a,^*	0.0026*	
*CYP3A4* (rs2242480)	GG (*n* = 14)	GA (*n* = 8)	AA (*n* = 1)	GA, AA (*n* = 9)			0.9438
C_min,ss_ (ng/mL)	35.03 ± 10.62	32.39 ± 8.95	33.7	32.53 ± 8.38	0.8400	0.5586	
C_max,ss_ (ng/mL)	326.07 ± 73.75	265.88 ± 41.14	258	265 ± 38.57	0.109	0.0332*	
AUC_τ_ (ng·h/mL)	2453.17 ± 567.87	2159.08 ± 323.73	2167.74	2160.04 ± 302.83	0.4014	0.1713	
Half-life (h)	14.46 ± 4.77	13.56 ± 2.55	12.66	13.46 ± 2.40	0.8343	0.5664	
CL/F (L/h)	8.57 ± 1.99	9.49 ± 1.75	9.23	9.46 ± 1.64	0.5582	0.2777	

*p<0.05.

^a^*p* < 0.05 between W and H.

^b^*p* < 0.05 between W and M.

HWE, Hardy-Weinberg equilibrium; C_max,ss_, maximum (peak) steady-state plasma drug concentration during a dosage interval; AUC_τ_, area under the drug concentration vs time curve within a dosing interval at a steady state; CL/F, apparent total body clearance of the drug from the plasma.

**TABLE 2:** *r*^2^ values of pair-wise linkage disequilibrium between eight single-nucleotide polymorphisms of *FMO3* and *CYP3A4*.

*r* ^2^	*FMO3* rs909530	*FMO3* rs1800822	*FMO3* rs2266780	*FMO3* rs2266782
*FMO3* rs1800822	0.2792			
*FMO3* rs2266780	^a^ **0.4889**	0.0564		
*FMO3* rs2266782	^a^ **0.4889**	0.0564	^b^ **1**	
*CYP3A4* rs2242480	0.0033	0.0017	0.0015	0.0015

a *r*^2^ > 0.3, strong linkage disequilibrium.

b *r*^2^ = 1, perfect linkage disequilibrium.

### Effect of *FMO3* Polymorphisms on Teneligliptin Pharmacokinetics

**FIGURE 1:**
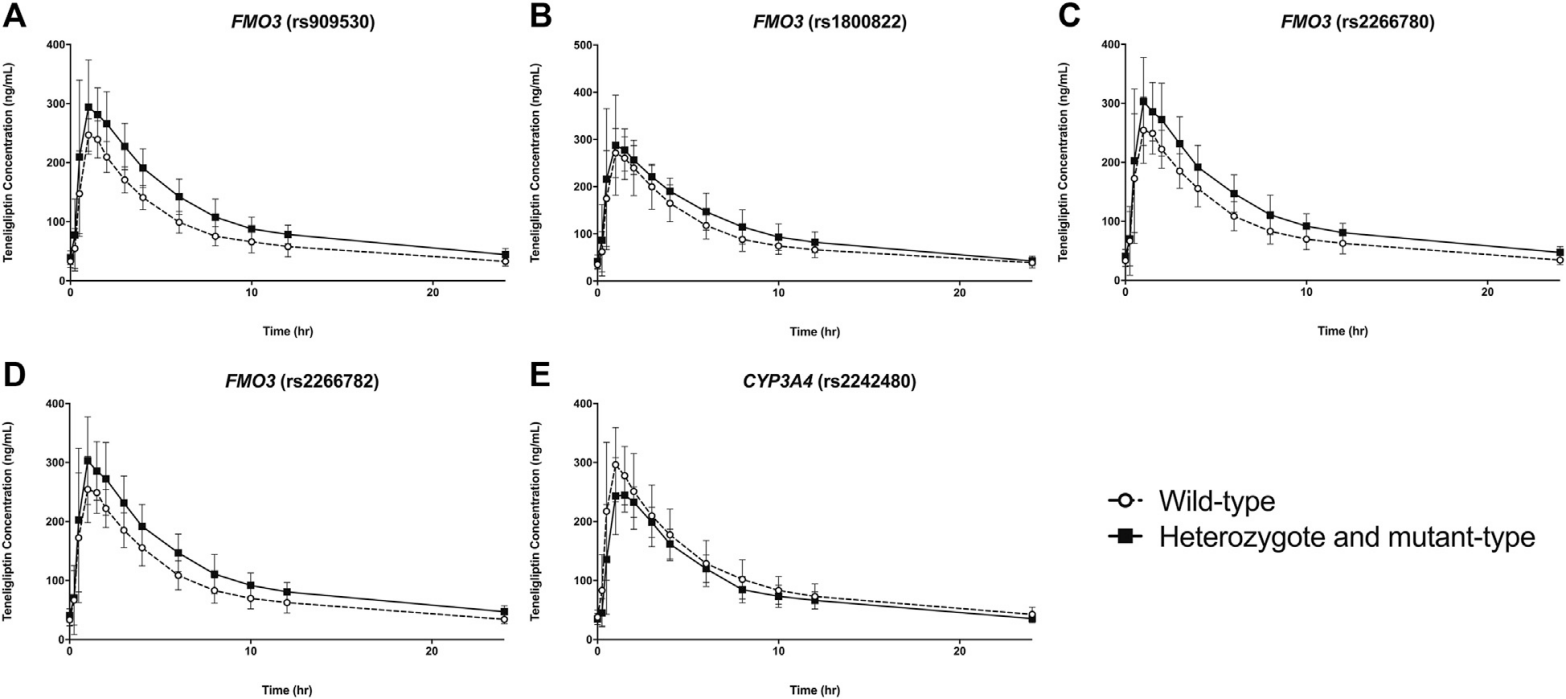
Plasma concentration vs time plots of teneligliptin according to polymorphisms of *FMO3* **(A–D)** and *CYP3A4* **(E)**. Data are represented as mean ± standard deviation.

**FIGURE 2:**
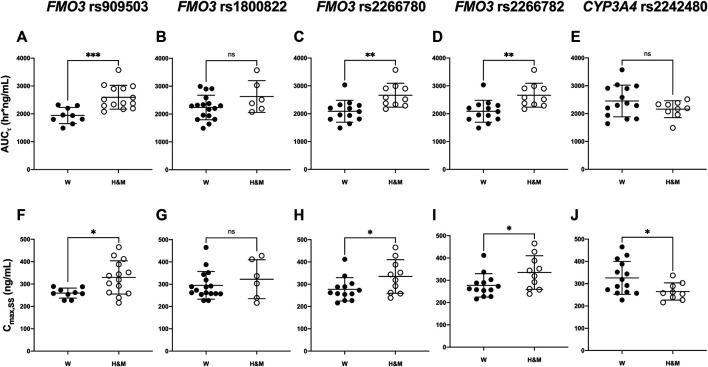
Comparison of pharmacokinetic parameters [area under the plasma concentration-time curve: **(A–E)** and C_max_: **(F–J)]** according to *FMO3* (rs909530, rs1800822, rs2266780, rs2266782) and *CYP3A4* (rs2242480) genotypes. Asterisks indicate significant differences between the two groups: wild (W) type *vs* heterozygous mutant (H) or mutant (M) types (***p ≤ 0.001,**p < 0.01,*p < 0.05).

The pharmacokinetic parameters of teneligliptin were comparatively analyzed according to *FMO3* polymorphisms. Subjects with rs909530 polymorphism exhibited gene dosage-dependent increases in C_max,ss_ (*p* = 0.0125) and AUC_τ_ (*p* = 0.0006) values and a decrease in oral clearance (CL/F) (*p* = 0.0002) (Table 1). The differences were highly marked in the recessive genetic model (wild-type vs heterozygous and homozygous mutant) (*p* = 0.0125, 0.0006, and 0.0002 for C_max,ss_, AUC_τ_, and CL/F, respectively; Table 1 and Figures 1, 2). Similarly, subjects with *FMO3* rs2266780/rs2266782 polymorphisms exhibited enhanced C_max,ss_ (*p* = 0.0426) and AUC_τ_ (*p* = 0.0031) values but decreased CL/F (*p* = 0.0026) values (Table 1; Figures 1, 2). *FMO3* rs1800822 polymorphism did not affect the pharmacokinetics of teneligliptin in this study. Also, *FMO3* polymorphisms did not affect the pharmacokinetics of teneligliptin sulfoxide (Supplementary Figure S1, Table S3).

### Effects of *CYP3A4* Polymorphism on Teneligliptin Pharmacokinetics

*CYP3A4* rs2242480 polymorphism did not affect the pharmacokinetics of teneligliptin and teneligliptin sulfoxide. The C_max,ss_ of teneligliptin significantly decreased in the recessive genetic model (wild-type: 326.07 ng/ml, heterozygous and homozygous mutant: 265 ng/ml; *p* = 0.0332; Table 1 and Figures 1, 2).

Additionally, *CYP3A4* rs2242480 polymorphism did not affect the pharmacokinetics of teneligliptin sulfoxide (Supplementary Figure S1, Table S3).

## Discussion

This study demonstrated that the rs909530, rs2266780, and rs2266782 (but not rs1800822) polymorphisms of *FMO3* and the rs2242480 polymorphism of *CYP3A4* affected the pharmacokinetics of teneligliptin in humans.

*FMO3* plays a crucial role in the disposition of its substrates in humans. The rs909530, rs2266780, and rs17565766 polymorphisms of *FMO3* affect the formation of sulindac sulfide, which is formed from the FMO3-mediated metabolism of sulindac (Park et al., 2014). In particular, rs909530 is the most important polymorphism affecting sulindac metabolism. One study reported that FMO3 rs909530 and rs2266780 polymorphisms influence sulindac metabolism in the Chinese population (Tang et al., 2017). The AUC and C_max_ values for sulindac disposition in subjects with homozygous mutants were 50 and 71% higher than those in subjects with wild-type alleles, respectively. In this study, the AUC_τ_ and C_max,ss_ values for teneligliptin in the rs909530 homozygous mutant group were 42 and 41% higher, respectively, than those in the wild-type group.

Among the four genetic polymorphisms of *FMO3* examined in this study, rs2266780 and rs2266782 exhibited complete linkage disequilibrium (*r* ^2^ = 1). Consistently, a previous study demonstrated that rs2266780 and rs2266782 exhibited a strong linkage disequilibrium (*r* ^2^ = 0.848–0.980) (Ren et al., 2017; Xu et al., 2017b). Similar to the effect of rs909530 on teneligliptin disposition, rs2266780/rs2266782 polymorphisms promoted gene dosage-dependent increase in teneligliptin exposure in this study. In contrast to the findings of this study, previous studies have demonstrated that rs909530 (but not rs2266782) is a key factor influencing the disposition of FMO3 substrates, including sulindac and tacrolimus (Park et al., 2014; Ren et al., 2017). This suggests that the polymorphic effect of FMO3 may be substrate-specific (Hisamuddin and Yang, 2007).

*FMO3* rs909530 is a synonymous mutation (N285N) that does not change the amino acid sequence (Waldman et al., 2011). Therefore, rs909530 may indirectly affect teneligliptin pharmacokinetics by modulating protein expression through different mechanisms (Johnson et al., 2005; Wang and Sadée, 2006; Sauna et al., 2007; Waldman et al., 2011). However, another synonymous polymorphism, rs1800822 (c.441C>T, S147S) did not affect teneligliptin pharmacokinetics. Similarly, tacrolimus disposition was affected by rs909530 but not by rs1800822 (Ren et al., 2017). A recent study has reported that six FMO3 SNPs, including rs909593, rs2266780, and rs2266783, were associated with significantly decreased protein abundance. However, these SNPs were not correlated with the mRNA expression of FMO3 (Xu et al., 2017a; Xu et al., 2017b) and may promote post-translational modifications (Xu et al., 2017a). We believe that the experimental evidence presented in this study supports the polymorphic effects of FMO3 on teneligliptin pharmacokinetics.

The effect of rs2242480 (CYP3A4*1G) polymorphism on CYP3A4 functions is controversial (Hu et al., 2007; Liu et al., 2017; Lolita et al., 2020). Current knowledge indicates that the effect of polymorphism on CYP3A4 activity is dependent on the substrate (Stresser et al., 2000). In this study, rs2242480 significantly decreased the Cmax, ss values of teneligliptin and markedly decreased teneligliptin exposure (AUC_τ_ values). This indicates that rs2242480 polymorphism regulates teneligliptin disposition by increasing CYP3A4 activity. Several studies have suggested the conflicting roles of CYP3A4*1G in CYP3A4 activity. CYP3A4*1G polymorphism enhances the disposition of CYP3A4 substrates, including atorvastatin (Gao et al., 2008), cyclosporin A (El-Shair et al., 2019), and tacrolimus (Tamashiro et al., 2017; Tang et al., 2019). In contrast, CYP3A4*1G polymorphism is reported to decrease the disposition of fentanyl (Yuan et al., 2015). However, we suggest that CYP3A4*1G polymorphism may increase the disposition of teneligliptin in a substrate-dependent manner (Stresser et al., 2000) based on the assumption that G-to-A substitution at IVS10 + 12 enhances the transcription of CYP3A4 *in vitro* (He et al., 2011).

Teneligliptin sulfoxide (M1) is the main metabolite formed from FMO3- and CYP3A4-mediated metabolism of teneligliptin (Nakamaru et al., 2014). The polymorphic *FMO3* genotype markedly increased teneligliptin exposure in this study. Hence, the blood levels of teneligliptin sulfoxide may be decreased due to dysfunctional *FMO3* activity. Although polymorphic *FMO3* genotypes decreased M1 exposure, the pharmacokinetic parameters of M1 were not significantly different between subjects with different *FMO3* polymorphisms. M1, which is the main metabolite formed from teneligliptin in humans (Nakamaru et al., 2014), is further metabolized into teneligliptin sulfone (M2) (Nakamaru et al., 2014). Therefore, the extent of metabolic conversion from M1 to M2 may have influenced the results. This study did not assess the formation of M2 as the enzymes involved in the formation of M2 have not been reported. However, M2 may be pharmacodynamically inactive based on the assumption that M1 is an inactive metabolite. Therefore, the quantification of M2 is not clinically essential (Halabi et al., 2013).

This study is associated with several limitations. In this study, only healthy male participants were recruited. Patients with T2DM exhibiting different *FMO3* and *CYP3A4* genotypes were not included to exclude other factors influencing teneligliptin disposition. In addition to teneligliptin, the study subjects may have undergone other drug therapies. Additionally, the pharmacodynamic effects of *FMO3* and *CYP3A4* genotypes on teneligliptin were not assessed in this study. As the study participants were healthy, they exhibited euglycemia. Therefore, the antidiabetic effects were not examined in this study. Further studies are needed to confirm the possible role of these polymorphisms in teneligliptin metabolism and their clinical effects.

In conclusion, the findings of this study suggest that the rs909530, rs2266780, and rs2266782 (but not rs1800822) polymorphisms of *FMO3* and the rs2242480 polymorphism of *CYP3A4* affect the pharmacokinetics of teneligliptin. Future studies must elucidate the effect of these polymorphisms in patients with diabetes and their influence on the pharmacodynamics of teneligliptin using a large population.

## Data Availability

The raw data supporting the conclusions of this article will be made available by the authors, without undue reservation.
