# Supplementary material for: Influence of FMO3 and CYP3A4 Polymorphisms on the Pharmacokinetics of Teneligliptin in Humans
Source: Front Pharmacol. 2021 Aug 26;12:736317. doi: 10.3389/fphar.2021.736317 (PMC8426351; doi:10.3389/fphar.2021.736317)
Supplement: Supplementary file 2 [file Table1.docx]

**Supplementary Table 1**. Oligonucleotide primers for *FMO3* and *CYP3A4* single nucleotide polymorphisms (SNPs) identification.

| SNP | Primer | Sequences | Size (bp) | PCR (Tm; °C) |
| --- | --- | --- | --- | --- |
| *FMO3 (*rs909530) | Forward | B 5’-TTGGGTCATTTTTTCCTTCCTTAT-3’ | 261 | 60 |
|  | Reverse | 5’-ACCCTGTTGCAAAGATTACACAGT-3’ |  |  |
|  | Sequencing | 5’-TTGCTGGGAGCTCAT-3’ |  |  |
| *FMO3* (rs1800822) | Forward | B 5’-CCACTGAAAGGGATGGTAAAAA-3’ | 125 | 60 |
|  | Reverse | 5’-AGCAGCTTAAATTTTGGCCTTAC-3’ |  |  |
|  | Sequencing | 5’-TGGGATACACATGATGTC-3’ |  |  |
| *FMO3* (rs2266780) | Forward | 5’-AGCATTCTGTGTGGCATTGT-3’ | 144 | 60 |
|  | Reverse | B 5’-AAGGAAGGGGTAGGCAAAACTAT-3’ |  |  |
|  | Sequencing | 5’-CGTGAAGGAATTCACAG-3’ |  |  |
| *FMO3* (rs2266782) | Forward | B 5’-ATGGTAAAAAAGAATCGGCTGTC-3’ | 132 | 60 |
|  | Reverse | 5’-TTTTGTCAGTTATGTGGCTAGCAG-3’ |  |  |
|  | Sequencing | 5’-GCCTTACCTGGAAAGGACT-3’ |  |  |
| *CYP3A4* (rs2242480) | Forward | B 5’-AAAAATTCTCCTGGGAAGTGG-3’ | 124 | 60 |
|  | Reverse | 5’-AGAAACTGCAGGAGGAAATTGA-3’ |  |  |
|  | Sequencing | 5’-CCAATAAGGTGAGTGGAT-3’ |  |  |

SNP, single nucleotide polymorphism; PCR, polymerase chain reaction; B, biotinylated at the end of the primer; T_m_, melting temperature
