# Supplementary material for: Influence of FMO3 and CYP3A4 Polymorphisms on the Pharmacokinetics of Teneligliptin in Humans
Source: Front Pharmacol. 2021 Aug 26;12:736317. doi: 10.3389/fphar.2021.736317 (PMC8426351; doi:10.3389/fphar.2021.736317)
Supplement: Supplementary file 3 [file Table2.docx]

**Supplementary Table 2.** Baseline demographic characteristics of study participants (*n*=23).

|  | Wild type | Heterozygote | Homozygous mutant | *p* value |
| --- | --- | --- | --- | --- |
| *FMO3 rs909530 (n)* | GG (*n* = 9) | GA (*n* = 10) | AA (*n* = 4) |  |
| Age (years) | 24 ± 2.35 | 25.5 ± 3.06 | 28 ± 6.16 | 0.1841 |
| Weight (kg) | 73.11 ± 6.72 | 65.55 ± 5 | 71.68 ± 8.14 | 0.0422^a^* |
| Height (cm) | 172.67 ± 4.58 | 172.50 ± 3.44 | 180 ± 1.41 | 0.0067^a,b,c^* |
| Body mass index (BMI, kg/m^2^) | 24.53 ± 2.19 | 22.04 ± 1.75 | 22.10 ± 2.32 | 0.033^a^* |
| *FMO3 rs1800822 (n)* | GG (*n* = 17) | GA (*n* = 5) | AA (*n* = 1) |  |
| Age (years) | 25 ± 2.94 | 26.4 ± 5.94 | 26 | 0.7534 |
| Weight (kg) | 69.93 ± 6.91 | 66.18 ± 5.77 | 80.5 | 0.1622 |
| Height (cm) | 173.06 ± 4.39 | 175.4 ± 4.83 | 180 | 0.2464 |
| Body mass index (BMI, kg/m^2^) | 23.37 ± 2.39 | 21.5 ± 1.34 | 24.85 | 0.2032 |
| *FMO3 rs2266780/2266782 (n)* | AA (*n* = 13) | AG (*n* = 9) | GG (*n* = 1) |  |
| Age (years) | 24.15 ± 1.99 | 27 ± 4.95 | 26 | 0.1933 |
| Weight (kg) | 71.86 ± 7.19 | 66.11 ± 5.89 | 71 | 0.1643 |
| Height (cm) | 173.23 ± 4.57 | 174 ± 4.42 | 181 | 0.2732 |
| Body mass index (BMI, kg/m^2^) | 23.94 ± 2.16 | 21.86 ± 2.09 | 21.67 | 0.0888 |
| *CYP3A4 rs2242480 (n)* | GG (*n* = 14) | GA (*n* = 8) | AA (*n* = 1) |  |
| Age (years) | 24.79 ± 2.97 | 26 ± 4.75 | 28 | 0.5879 |
| Weight (kg) | 67.86 ± 6.44 | 73.36 ± 6.98 | 63.2 | 0.1336 |
| Height (cm) | 173.14 ± 4.74 | 174.75 ± 4.59 | 177 | 0.5952 |
| Body mass index (BMI, kg/m^2^) | 22.66 ± 2.30 | 24.01 ± 2.04 | 20.17 | 0.1886 |

Mean ± SD for continuous variables. **p*<0.05, ^a^*p*<0.05 between W and H, ^b^*p*<0.05, between W and M, ^c^*p*<0.05, between H and M.
