# Supplementary material for: Influence of FMO3 and CYP3A4 Polymorphisms on the Pharmacokinetics of Teneligliptin in Humans
Source: Front Pharmacol. 2021 Aug 26;12:736317. doi: 10.3389/fphar.2021.736317 (PMC8426351; doi:10.3389/fphar.2021.736317)
Supplement: Supplementary file 4 [file Table3.docx]

**Supplementary Table 3.** Effect of *FMO3* and *CYP3A4* polymorphisms on the pharmacokinetic parameters of teneligliptin sulfoxide.

| Parameter | Wild type (W) | Heterozygous (H) | | Homozygous mutant (M) | H & M | *p* value | | |  |
| --- | --- | --- | --- | --- | --- | --- | --- | --- | --- |
|  |  |  | |  |  | | W vs H vs M | W vs H & M | |
| FMO3 (rs909530) | GG (n = 9) | | GA (n = 10) | AA (n = 4) | GA, AA (n = 14) | |  |  | |
| C_max,ss_ (ng/mL) | 52.87 ± 11.13 | | 58.11± 22.72 | 39.68 ± 13.25 | 52.84 ± 11.13 | | 0.2317 | 0.9976 | |
| AUC_τ_ (ng·h/mL) | 431.88 ± 198.10 | | 794.98 ± 721.63 | 303.67 ± 146.78 | 654.61 ± 646.95 | | 0.1759 | 0.3307 | |
| *FMO3* (rs1800822) | GG (*n* = 17) | | GA (*n* = 5) | AA (*n* = 1) | GA, AA (*n* = 6) | |  |  | |
| C_max,ss_ (ng/mL) | 51.21 ± 13.54 | | 61.94 ± 29.31 | 35.30 | 57.50 ± 28.38 | | 0.3209 | 0.4751 | |
| AUC_τ_ (ng·h/mL) | 645.67 ± 589.16 | | 380.95± 96.53 | 170.29 | 345.84 ± 121.87 | | 0.4722 | 0.2361 | |
| *FMO3* (rs2266780/ rs2266782) | AA (*n* = 13) | | AG (*n* = 9) | GG (*n* = 1) | AG, GG (*n* = 10) | |  |  | |
| C_max,ss_ (ng/mL) | 56.05 ± 19.51 | | 50.62 ± 15.52 | 31.30 | 48.69 ± 15.86 | | 0.3893 | 0.3427 | |
| AUC_τ_ (ng·h/mL) | 392.86 ± 184.75 | | 861.62 ± 733.09 | 189.63 | 794.42 ± 723.09 | | 0.0848 | 0.0667 | |
| *CYP3A4* (rs2242480) | GG (*n* = 14) | | GA (*n* = 8) | AA (*n* = 1) | GA, AA (*n* = 9) | |  |  | |
| C_max,ss_ (ng/mL) | 51.63 ± 17.18 | | 53.61 ± 21.23 | 63.90 | 54.76 ± 20.15 | | 0.8114 | 0.6943 | |
| AUC_τ_ (ng·h/mL) | 645.31 ± 647.18 | | 445.74 ± 228.31 | 451.25 | 446.35 ± 213.57 | | 0.6932 | 0.3860 | |

C_max,ss_, maximum (peak) steady state plasma drug concentration during a dosage interval; AUC_τ_, area under all concentration-time curves within a dosing interval at steady state.
